# Recent Experiments with Mallein—A Lymph Made from Cultures of the Bacillus of Glanders

**Published:** 1891-09

**Authors:** Leonard Pearson


THE JOURNAL

OF

COMPARATIVE MEDICINE AND

VETERINARY ARCHIVES.

VOL. XII. SEPTEMBER, 1891. NO. 9.

### RECENT EXPERIMENTS WITH MALLEIN—A LYMPH MADE FROM CULTURES OF THE BACILLUS OF GLANDERS.

BY LEONARD PEARSON, B.S., V.M.D.

Hellmann, of the Pasteur Institute, in St. Petersburg, read a paper before a Scientific Society of that city, late in the month of March, in which he described some experiments made by him, with an aqueous extract of potato cultures of the glanders bacillus. He found that subcutaneous injections of 1 c.c. of this fluid, in glanderous horses, was sufficient to cause an elevation of the temperature to 40°C. (104°F.), and higher; weakness, loss of appetite, local reaction at the point of injection and increased discharge from the nose. Injections into healthy horses, or horses suffering from other diseases than glanders, are said to be followed by no reaction. In one or two cases injections in which glanders had not been diagnosed were followed by febrile reactions, but the subsequent autopsies showed the presence of the characteristic nodules of the disease. These experiments were made on cavalry horses of the Russian army.

Hellmann made his Mallein by floating off potato cultures of glanders bacillus with water, adding a little glycerine to the mixture and heating it, at first to 40°C., then to 80°C. and, finally, sterilizing at 150°C.

Dr. Kalming, in the Veterinary School of Dorpat, Russia, has also extracted a substance from glanders cultures which, when injected into glanderous horses, causes a characteristic febrile reaction, but no effect on healthy animals. Recent dispatches from Dorpat report the sad news that Dr. Kalming, in the course of his experiments, infected himself with glanders and has since died from this disease. Before the publication of any of this work I had commenced, the first of March, a series of experiments to determine whether glanders cultures developed a toxine, or toxalbumine, which would have an action similar to that of tuberculine. My Mallein was prepared as follows :

Sterilized glycerine bouillon; made of meat extract, peptone, sodium chloride and glycerine, was inoculated with virulent (second generation) glanders bacilli, from potato cultures, and kept fourteen days in an incubator at 36.5°C. (The bacilli grew luxuriantly.) The cultures were now heated at 80°C. for several hours, to kill the bacilli and condense the fluid; they were next filtered, through Kitasato's porcelain filter, and on three successive days heated, for twenty minutes each time, in moving steam (100°C.). The lymph made in this way is a perfectly clear fluid, a little thicker and darker than the original bouillon. Glanderous and healthy guinea-pigs were injected with doses of 0.25-2.0 c.c. of this substance and the diseased animals showed, without exceptions, both a general and local reaction. The former was shown by depression, loss of appetite and fever; the latter by redness and swelling, both of the inoculation wound and the point of injection. The healthy animals did not react unless the dose was very large.

First experiment.* Male guinea-pig, inoculated with virulent glanders March 13th. Daily rectal temperature of 39.2°C. March 24th, injected in the flank (knee fold) with 0.5 c.c. of the lymph.

| | |
|---|---|
| Temperature shortly before the injection, | 39.2 |
| " after 4 hours, . . | 39.2 |
| " " 7 " . . . | 39.4 |
| " " 10 " . . | **39.6** |
| " " 12 " . . . | **39.8** |
| March 25th, A. M.. . . . . | 39.2 |
| " 26th, " . . . . . | 38.4 |

Although the animal was almost dead an injection of 0.3 c.c. was made.

| | |
|---|---|
| After 6 hours, . . . . . . | 37.8 |
| " 10 " . . . . . . | dead |

The autopsy showed glanderous processes in the spleen and testicles.

*These tables are also used in an article, by me, on the same subject, in the *Zeitschrift für Veterinärkunde*, Vol. iii. No. 5, 1891.

Second experiment. Male guinea-pig, inoculated March 20. Daily rectal temperature of 39-39.2°C. March 24th, injected 0.25 c.c. of the lymph.

| | | |
|---|---|---|
| Temperature shortly before the injection, | | 39.0 |
| " | after 4 hours, . . | 39.0 |
| " | " 7 " . . . | ***39.3*** |
| " | " 10 " . . | ***39.4*** |
| " | " 12 " . . . | ***40.1*** |
| March 25th, A. M., | . . . . | 38.1 |
| " 26th, " | . . . . . | 38.9 |

A second injection of 0.4 c.c. was now made.

| | | |
|---|---|---|
| Temperature after 6 hours, | . . | ***39.3*** |
| " | " 11 " . . . | ***39.8*** |
| March 27th, A. M., | . . . . | 39.1 |
| " 28th, " | . . . . . | 39.0 |

Third experiment. Male guinea-pig, inoculated April 16th. Daily rectal temperature 40.0-40.1-40.2°C. April 21st, injected 0.5 c.c.

| | | |
|---|---|---|
| Temperature after 6 hours, | . . | 40.1 |
| " | " 9 " . . . | ***40.6*** |
| " | " 12 " . . | ***40.5*** |
| April 22d. | morning, . . . | 40.3 |
| " | afternoon, . . . | 40.2 |
| " | evening, . . . | 39.4 |
| April 23d. | morning, . . . | 38.6 |
| " 24th, | . . . . . . . | 39.1 |

A second injection of 0.5 c.c. was now made.

| | | |
|---|---|---|
| Temperature after 6 hours, | . . | ***39.4*** |
| April 25th, | morning, . . . | 38.3 |
| " | afternoon, . . | dead |

The autopsy revealed glanders nodules in the spleen, and glanders bacilli in the blood.

Fourth experiment. Male guinea-pig, inoculated April 16th. Daily temperature 39.6° to 39.7°C.

| | | |
|---|---|---|
| April 21st., injected 0.5 c.c. | Temp. | 39.6 |
| " | after 6 hours. . . . | 39.7 |
| " | 9 " . . . | 39.8 |
| " | 12 " . . . | ***40.2*** |

CONTROLE EXPERIMENTS.

First experiment. Healthy male guinea-pig, for several days before the injection the temperature ranged from 38.6-38.9°C.

| | | |
|---|---|---|
| April 21st. | Temperature, . . . | 38.7 |
| " | Injected 0.5 c.c. | |

| | |
|---|---|
| Temperature after 6 hours, . . | 38.7 |
| " " 9 " . . . . | 38.6 |
| " " 12 " . . . . | 38.7 |
| April 22, . . . . . . . | 38.7 |
| " 23, . . . . . . | 39.0 |
| " 24, . . . . . . | 39.3 |
| Injected 0.5 c.c. | |
| Temperature after 6 hours, . . | 38.9 |
| " " 9 " . . . . | 39.0 |
| April 25th, . . . . . | 39.0 |

Second experiment. Healthy male guinea-pig. Before the injection the temperature varied between 38.7 and 39.0°C.

| | |
|---|---|
| April 21st, injected 0.5 c.c. Temp. | 39.0 |
| Temperature after 8 hours, . . | 39.0 |
| " " 9 " . . . . | 38.9 |
| " " 12 " . . | 38.9 |
| April 22d, A. M., . . . . . | 39.0 |
| " 23d, " . . . . . | 38.9 |

Third experiment. Healthy male guinea-pig. Temperature before the injection between 38.6-38.8°C.

| | |
|---|---|
| April 7th, injected 2.0 c.c. Temp. | 38.8 |
| Temperature after 8 hours, . . | 39.3 |
| " " 12 " . . . . | 39.4 |
| " 8th, A. M., . . . . . | 39.3 |
| " 9th, " . . . . . | 39.3 |
| " 10, " . . . . . . | 39.2 |
| " 11, " . . . . . | 39.0 |

Fourth experiment. Healthy male guinea-pig. Temperature, before the injection, ranged from 38.2 to 38.6°C.

| | |
|---|---|
| April 2d, injected 2.0 c.c. Temp. | 38.2 |
| Temperature after 8 hours, . . | 38.4 |
| " " 12 " . . . . | 39.0 |
| April 3d, A. M., . . . . . | 38.5 |
| " 4th, " . . . . . | 38.8 |
| " 5th, " . . . . . | 38.6 |

These experiments show that the action of mallein on healthy and glanderous guinea-pigs differ. Injections of doses of 0.25 to 0.5 in diseased animals, causes a decided local and general reaction, while these doses in healthy animals produce either no change whatever or at most, a slight redness of the point of injection. Large doses, on the contrary,(2.0 c.c.), cause a transitory fever. The resemblance, in these respects, between mallein and tuberculine is quite noticeable. I could not see, in those cases in which repeated injections were made, that the mallein had the least healing effect on the glanderous process. All of the inoculated animals died of generalized glanders. To what extent immunity can be produced by mallein is, at present, impossible to say. I shall endeavor to make this an object of further experiment.

The above experiments were made in the Laboratory of the Veterinary School of the Prussian Army, and I wish to use this opportunity to thank its Director, Chief Veterinarian Hall, for his kindness and favors to me during their progress.

Since the above was written an article has appeared in the *Berliner Thierarzliche Wochenschrift* (No. 29, 1891), by Preusse, of Danzig, giving the results of some experiments made by him with mallein. He used a fluid made by extracting potato cultures with equal parts of glycerine and water, and made his experiments on horses. Six glanderous and two healthy horses were injected with doses of from 0.1 to 0.3 c.c.; the former reacted strongly in from six to twelve hours, while the latter showed no change. The reaction was both local and general; the temperature rose, in some cases, to 40.1 and 40.4°C. All of the horses were killed and the post-mortem examinations confirmed the ante-mortem diagnoses in each instance.

It is to be hoped that we will have here a medium for diagnosing glanders, as safe as the tuberculine is for diagnosing tuberculosis of cattle. The latest authentic report shows success in about 98 per cent. of the cases of tuberculosis.

